# Impact of Body Image Perception on Behavioral Outcomes in Chinese Adolescent and Young Adult Survivors of Sarcoma

**DOI:** 10.1002/cam4.70320

**Published:** 2024-12-02

**Authors:** Yihui Wei, Chung Tin Ma, Michael Can Heng Li, Keary Rui Zhou, Herbert Ho Fung Loong, Kwok Chuen Wong, Chi Kong Li, Yin Ting Cheung

**Affiliations:** ^1^ School of Pharmacy, Faculty of Medicine, The Chinese University of Hong Kong Hong Kong SAR China; ^2^ Department of Clinical Oncology, Faculty of Medicine The Chinese University of Hong Kong Hong Kong SAR China; ^3^ Department of Orthopaedics and Traumatology Prince of Wales Hospital Hong Kong SAR China; ^4^ Department of Paediatrics, Faculty of Medicine The Chinese University of Hong Kong Hong Kong SAR China; ^5^ Department of Paediatrics & Adolescent Medicine Hong Kong Children's Hospital Hong Kong SAR China; ^6^ Hong Kong Hub of Paediatric Excellence The Chinese University of Hong Kong Hong Kong SAR China

**Keywords:** adolescents and young adults, behavioral outcomes, body image dissatisfaction, sarcoma survivors

## Abstract

**Purpose:**

To identify the prevalence and predictive factors of body image dissatisfaction among Chinese adolescent and young adult (AYA) survivors of sarcoma and to evaluate its associations with behavioral outcomes.

**Methods:**

In total, 116 AYA survivors (response rate: 88%; 48.3% female; mean age 28.2 years, SD = 8.2 years) of osteosarcoma (49.1%) or soft‐tissue sarcoma (50.9%) were recruited from an academic hospital. The survivors self‐reported their perceived body image using the Body Image Scale. Behavioral outcomes were assessed using DSM‐oriented scales of the ASEBA Adult Self‐Report checklist. Multivariable linear regression was conducted to identify predictors of body image perception and to investigate the association between body image dissatisfaction and behavioral outcomes (adjusted for clinically relevant variables and corrected for false discovery rate). Mediation analyses were performed to examine the mediating effects of body image perception between clinical, treatment, or socioenvironmental risk factors and behavioral outcomes.

**Results:**

At 15 years post‐cancer diagnosis, one‐third of the cohort (39.7%) reported dissatisfaction with their body image. The significant risk factors associated with body image dissatisfaction were being female (standardized coefficient estimate [Est] = 0.21, SE = 0.10; *p* = 0.047), surgery treatment (Est = 0.19, SE = 0.09; *p* = 0.046), and worse family functioning (Est = 0.27, SE = 0.10, *p* = 0.013). Body image dissatisfaction was associated with symptoms of depression (Est = 0.30, SE = 0.10; *p* = 0.005) and avoidant personality problems (Est = 0.37, SE = 0.11; *p* < 0.001). Negative body image perception significantly mediated the association between poor family functioning and avoidant personality problems (proportion‐mediated 26.3%, *p* = 0.038).

**Conclusion:**

Body image dissatisfaction was associated with more significant internalizing symptoms of depression, anxiety, and avoidant personality. A negative body image perception may mediate the association between poor family functioning and psychological distress among adult survivors. The provision of psychosocial intervention early during the cancer care continuum may mitigate the negative impact of body image distress in Chinese AYA survivors.

## 1 Introduction

Osteosarcoma and soft tissue sarcoma (STS) are relatively rare cancers among adolescents and young adults (AYAs). Improved treatment strategies for childhood osteosarcoma and STS that involve surgery, chemotherapy, and radiation have led to increased survival rates [1, 2]. However, survivorship comes at the cost of developing myriad adverse effects that can affect the survivor's quality of life and functional outcomes later in life [1]. Due to intensive surgical and radiological treatments, survivors of STS may experience body image dissatisfaction and negative body changes such as scarring, amputation, and disfigurement, and these issues may persist into long‐term survivorship [3].

Most studies on body image perception have been conducted in adult patients with breast, head and neck, and gynecological cancers [4, 5, 6, 7]. Much less attention has been paid to body image perception among survivors of sarcoma. Embarrassment and concerns about their altered physical appearance were common themes identified in a qualitative study on sarcoma survivors' perspectives on their body image after resection/limb salvage surgery [8]. One recent study also showed that patients with bone tumors reported worse scores than healthy controls for physical categories of quality of life, as well as for functional and esthetic aspects of body image [9]. This may reflect persistent body image concerns and negative perceptions of physical changes among survivors of sarcoma, especially those with scars or highly visible disfigurement.

According to the self‐discrepancy theory, the discrepancy between the actual and ideal body image can lead to psychological distress and adverse behavioral outcomes [10]. Surgical treatment, weight changes, and socioenvironmental factors during the cancer experience may lead to body image concerns in survivors [3]. When negative perceptions of body image are internalized, they may manifest as behavioral problems, such as social withdrawal, anxiety, and depression, during survivorship [11, 12]. AYAs with cancer may be particularly vulnerable to body image disturbance, as they are often affected by pressure from the media, and their family members and peers [13, 14]. As a young individual's perception is often heavily driven by the attachment patterns which arise from relationships between parents and siblings, family functioning may also represent a crucial factor in the development of body image perception [15, 16]. Among adult survivors of childhood cancer, it has been reported that body image dissatisfaction mediates the association between treatment‐related scarring/disfigurement and psychological distress, regardless of sex [12]. From a developmental perspective, the impact of scarring and/or disfigurement may be particularly detrimental when it is acquired in or persists through adolescence and young adulthood, because these periods are critical for establishing social and sexual identities [17, 18].

Currently, most reports of body image perception in AYA survivors of cancer relate to Western populations. In Norway, a qualitative study involving sarcoma survivors has reported concerns about visible differences and functional impairments [19]. Another Australian study also revealed negative mental and functional experiences resulting from body image issues among sarcoma survivors [8]. To highlight, one cross‐cultural study showed that perceptions regarding body image can differentially impact Asian American, African American, and Caucasian women's experiences with cancer [20]. Other than peer influence, cultural beauty norms might especially be affected by parents, family, and peers in collectivistic societies such as Chinese populations [21]. Consequently, body image dissatisfaction might have more detrimental impact on behavioral and psychological outcomes in Chinese cancer survivors. Most body image reports in the Chinese cancer population are focused on survivors of breast cancer [22, 23, 24], and none has been conducted on survivors of sarcoma, who often undergo extensive surgery and radiation as part of their curative treatment.

The objectives of this study were (1) to identify clinical, treatment, socio‐environmental, and lifestyle factors associated with body image perception in Chinese AYA survivors of sarcoma and (2) to evaluate the association between body image perception and behavioral outcomes. We also included an exploratory objective to examine the mediating effects of body image perception on the association between clinical, treatment, socio‐environmental, lifestyle factors, and behavioral outcomes in this population.

## 2 Material and Methods

### 2.1 Study Design

This prospective, observational study was conducted at the long‐term survivors' clinics of the Department of Orthopedics and Traumatology and the Department of Pediatrics of the Prince of Wales Hospital in Hong Kong. Eligible participants were recruited through consecutive sampling. Between June 2020 and May 2022, the study investigators obtained the list of patients who were scheduled to attend follow‐up consultations at the clinics. Patients were then screened for eligibility using the in‐house electronic patient record system (Clinical Management System). All eligible patients who subsequently attended the long‐term follow‐up clinic were invited to participate in the study. The study was approved by the Joint Chinese University of Hong Kong–New Territories East Cluster Clinical Research Ethics Committee (Ref: 2018.131). Participants who were 18 years old or over provided written informed consent. For those who were under 18, written informed consent was provided by their parents.

### 2.2 Study Population

Survivors were eligible for the study if they were aged 15 to 39 years old and had been diagnosed with osteosarcoma or STS. Consistent with the definition adopted by other survivorship studies [25, 26, 27, 28, 29, 30], the participant had to have survived for at least 5 years post‐diagnosis and 2 years after completion of cancer treatment. Survivors who had any pre‐existing developmental or behavioral disorders diagnosed by a physician (as identified from the in‐house electronic patient record system) were excluded.

### 2.3 Body Image Perception

Body image perception was evaluated using the 10‐item Body Image Scale (BIS) [31], which was developed to assess changes in body image in patients with cancer. The scale comprises (1) cognitive items, which relate to the cognitive appraisals individuals make of their appearance (e.g., dissatisfaction with one's appearance, or with scarring), (2) affective items, which relate to the emotions associated with one's appearance (e.g., feeling feminine, feeling attractive), and (3) behavioral items, which relate to how individuals behave toward their body (e.g., finding it hard to look at oneself naked, avoiding people because of one's appearance) [31]. Each item is assessed using a 4‐point scale from “not at all” to “very much.” A higher score is indicative of higher dissatisfaction with body image. Patients who reported a total score of ≥ 10 points are categorized as “dissatisfied with their body image” [7, 32].

The BIS is chosen to assess body image perception in this cohort as a recent systematic review found that it is one of the most widely used tools to evaluate body image perception in diverse populations of cancer patients [33], and it has strong evidence supporting its structural validity, internal consistency, and reliability. The Chinese version of the BIS has been previously translated from English according to the guidelines stipulated by the Translation and Cultural Adaptation–Principles of Good Practice [34], including forward and backward translations, pilot testing, and cognitive briefing, in Chinese patients with cancer [35]. As no study has yet validated the BIS in AYA cancer survivors in Hong Kong, we conducted an exploratory analysis to evaluate the internal consistency and corrected item‐total correlation of the items using Cronbach's *α* and Pearson's correlation coefficient, respectively [36]. The results are presented in Table S1. Cronbach's *α* was 0.91 for the overall BIS and ranged from 0.75 to 0.85 for the three domains, suggesting satisfactory internal consistency. The corrected item‐to‐scale correlation coefficient ranged from 0.62 to 0.82; no problematic or inconsistent item was identified.

### 2.4 Behavioral Outcomes

Behavioral functioning was evaluated using the traditional Chinese version of the Achenbach System of Empirically Based Assessment (ASEBA) Adult Self‐Report (ASR) checklist [37]. The primary outcomes of interest were the DSM‐oriented scales for Depressive Problems, Anxiety Problems, Somatic Problems, Avoidant Personality Problems, and Antisocial Personality Problems.

This scale was selected as the DSM‐oriented scales consist of items that international experts have rated as being consistent with DSM criteria for disorders that are defined mainly in terms of behavioral, emotional, social, and thought problems [37]. The Chinese version of the ASR has been used and validated in the Chinese general population [38], and used to evaluate behavioral outcomes in AYA survivors of cancer in the local population [26, 39]. All behavioral outcomes were transformed into age‐adjusted *T*‐scores (mean = 50; standard deviation [SD] = 10). All *T*‐scores were scaled such that a higher score indicated worse behavioral functioning. To estimate the prevalence of impairments within the study sample, impairment was defined as a score worse than 1.5 SD of the age‐adjusted *T*‐score, a definition that has been adopted by previous literature [26, 29].

### 2.5 Predictors of Body Image Perception

Predictors of body image perception were determined a priori based on a literature review. Sociodemographic predictors included sex [5], age at follow‐up [4, 5], highest educational attainment [4], and employment status [4, 5, 40]. Body mass index (BMI) was calculated and classified into three categories, including underweight (BMI < 18.5 kg/m^2^), normal‐weight (BMI: 18.5–23 kg/m^2^), and overweight/obesity (BMI ≥ 23 kg/m^2^), based on the World Health Organization classification for Asia‐Pacific populations [41]. Clinical predictors were cancer diagnosis, age at cancer diagnosis, history of relapse, presence of chronic health conditions, and weight status [42, 43, 44, 45]. Information on chronic health conditions (defined as a pre‐existing comorbidity or a health condition with an onset after the completion of cancer treatment, as diagnosed by a physician) was collected as it is reported to be associated with body image perception and behavioral outcomes in AYA survivors of cancer [44]. Treatment predictors included surgery, radiation, and chemotherapy for the treatment of the primary cancer/tumor and metastasis (if any). To assess the limb functioning after treatment among survivors with osteosarcoma, the Musculoskeletal Tumor Society (MSTS) scoring was calculated, including items of pain, function, emotional acceptance, use of any external support, walking ability, and gait alteration. Each item was rated in a scale of 0 to 5, and the total score ranged from 0 to 30, with higher scores indicating better limb functioning [46]. Both the clinical information and the treatment information were abstracted from the Clinical Management System, an electronic health data repository of the public healthcare system in Hong Kong.

The socio‐environmental predictors were family functioning [15, 16] and physical activity [47, 48]. Family functioning was assessed using the validated Chinese Family Assessment Instrument (CFAI) [49, 50]. The CFAI is a 33‐item tool that measures the domains of mutuality, communication and cohesiveness, conflict and harmony, parental concern, and parental control. The item scores are summed to yield total scores ranging from 33 to 165, and a higher score represents poorer family functioning. The participants self‐reported their physical activity level using the validated Chinese University of Hong Kong: Physical Activity Rating for Children and Youth (CUHK‐PARCY) [51, 52], which uses an 11‐point scale (0 to 10) to evaluate the level, intensity, and frequency of physical activity based on the metabolic equivalent of task score. The CUHK‐PARCY has been validated and has been used to measure physical activity in Chinese survivors of childhood cancer [26, 52]. A score of 0–3 is regarded as indicating a low activity level, while 4–6 represents moderate activity and 7–10 indicates a high activity level.

### 2.6 Statistical Analysis

The sample characteristics and outcome measures were summarized using descriptive statistics for the overall cohort and stratified by body image perception.

To identify factors associated with body image perception, the Mann–Whitney *U*‐test or Kruskal–Wallis test (for continuous variables) and chi‐square test (for categorical variables) were used to compare the sociodemographic, clinical, and behavioral characteristics of survivors who were satisfied versus dissatisfied with their body image. Univariate and multivariable general linear modeling was conducted to identify the factors associated with BIS total score (as a continuous variable). The Kruskal‐Wallis test was used to compare the continuous BIS total scores in survivors who have received different types of surgery (i.e., amputation, resection with allograft, resection with prosthesis, and resection in other sites).

To estimate the prevalence of impairments within the study samples, impairment was defined as a *T*‐score of > 65 (i.e., 1.5 SD worse than the population norms). *T*‐scores of behavioral outcomes were compared between survivors satisfied or dissatisfied with their body images using *t*‐test. General linear modeling was also conducted to evaluate the association between body image perception and behavioral outcomes, adjusting for clinically relevant covariates that are associated with behavioral outcomes in survivors of cancer: age [4, 5], sex [5, 11], cancer diagnosis [11], surgery [53], radiation [11], and weight status [5, 11]. For survivors of osteosarcoma, a subgroup analysis was conducted to include MSTS score as an additional covariate in the model as some studies have shown that functional status can affect body image perception and behavioral outcomes in cancer patients who had undergone surgical treatment [54, 55]. Standardized coefficient estimate (Est) and standard error (SE) were used to quantify the effect size of the associations. The multivariable analysis was adjusted for false discovery rate [56].

To address the exploratory objective, mediation analyses were performed to examine the mediating effects of body image between clinical, treatment, or socioenvironmental risk factors and behavioral outcomes. To ensure that the model was meaningful and to reduce redundancy, only risk factors that were significantly associated with body image in the multivariable models were included in the mediation analyses. Separate mediation models were run for each behavioral outcome with continuous BIS score as the mediator. Survivors' age, sex, cancer diagnosis, radiation, and weight status, which were clinically associated with behavioral outcomes [5, 11, 53], were included as covariates within each mediation model. Proportion‐mediated, unstandardized point estimates and bootstrapped 95% CIs (BCCI) for the total indirect effect and specific indirect pathways were estimated [57, 58].

The significance threshold was set at *p* < 0.05. All statistical analyses were performed using SAS (SAS 9.4, SAS Institute, Cary, NC, USA) and were two‐tailed.

## 3 Results

### 3.1 Characteristics of Study Cohort

A total of 132 survivors fulfilled the inclusion criteria, of whom 7 survivors were uncontactable (*n* = 2), had defaulted follow‐up appointment (*n* = 4), or had relocated to another country (*n* = 1). Subsequently, 125 were invited to participate in the study, of whom 118 survivors have provided informed consent. Two survivors dropped out and did not complete the assessment because of competing commitments. Finally, 116 survivors completed the assessments and were included in the analysis, with the response rate of 88% (Figure S1).

The average age at evaluation was 28.2 (SD = 8.2) years, and 48.3% were female. Approximately half of the survivors (50.9%) had been diagnosed with STS and 57 (49.1%) had been diagnosed with osteosarcoma. The average length of time since diagnosis was 14.9 (SD = 7.6) years. The majority of the osteosarcoma survivors had received tumor resection with prosthesis (35.7%) or allograph reconstruction (17.9%), while the majority of the STS survivors had received local tumor resection (43.7%). For the 57 survivors of osteosarcoma, the mean MSTS score was 27.3 points out of 30 (SD: 3.6, range 13 to 30), indicating relatively good limb functioning and low pain levels in majority of the survivors. Table 1 summarizes characteristics of the study population.

**TABLE 1:** Characteristics of overall participants and grouped by satisfaction of body image.

Characteristics	Overall *n* = 116	Dissatisfied *n* = 46	Satisfied *n* = 70	*p*
	*n*	*n* (%)^a^	*n* (%)^a^	
Sex				0.937^b^
Male	60	24 (40.0)	36 (60.0)	
Female	56	22 (39.3)	34 (60.7)	
Age at interview mean [SD]	28.2 [8.2]	30.8 [7.3]	26.5 [8.3]	**0.005** ^c^
> 15–18 years	13	1 (7.7)	12 (92.3)	**0.002** ^b^
≥ 18–30 years	58	19 (32.8)	39 (67.2)	
> 30 years	45	26 (57.8)	19 (42.2)	
Age of cancer diagnosis mean [SD]	13.3 [7.2]	14.4 [5.8]	12.5 [8.0]	0.164^c^
< 15 years	76	29 (38.2)	47 (61.8)	0.683^b^
≥ 15–20 years	27	12 (44.4)	15 (55.6)	
≥ 20–30 years	8	4 (50.0)	4 (50.0)	
> 30 years	5	1 (20.0)	4 (80.0)	
Years from diagnosis mean [SD]	14.9 [7.6]	16.4 [7.3]	14.0 [7.6]	0.097^c^
≤ 5 years	16	7 (43.8)	9 (56.3)	0.075^b^
> 5–10 years	16	2 (12.5)	14 (87.5)	
> 10–20 years	53	21 (39.6)	32 (60.4)	
> 20 years	31	16 (51.6)	15 (48.4)	
Education level				**0.040** ^b^
Secondary school or below	42	11 (26.2)	31 (73.8)	
Post‐secondary school or above	70	32 (45.7)	38 (54.3)	
Employment status				**0.001** ^b^
Employed	81	40 (49.4)	41 (50.6)	
Not employed/Students	35	6 (17.1)	29 (82.9)	
Clinical characteristics				
Cancer				
Soft‐tissue sarcoma	59	21 (35.6)	38 (64.4)	0.363^b^
Osteosarcoma	57	25 (43.9)	32 (56.1)	
Subtypes (for soft‐tissue sarcoma only)				NA
Rhabdomyosarcoma	5	2 (40.0)	3 (60.0)	
Ewing sarcoma	11	4 (36.4)	7 (63.6)	
Liposarcoma	2	1 (50.0)	1 (50.0)	
Synovial sarcoma	5	2 (40.0)	3 (60.0)	
Clear cell sarcoma	4	0	4 (100)	
PNET	3	1 (33.3)	2 (66.7)	
Others	29	11 (37.9)	18 (62.1)	
Tumor site (for soft‐tissue sarcoma)				NA
Lower extremity	18	6 (33.3)	12 (66.7)	
Upper extremity	6	3 (50.0)	3 (50.0)	
Abdomen/pelvis	17	6 (35.3)	11 (64.7)	
Head and neck	10	2 (20.0)	8 (80.0)	
Chest	8	4 (50.0)	4 (50.0)	
Tumor site (for osteosarcoma)				NA
Femur	32	17 (53.1)	15 (46.9)	
Tibia	14	2 (14.3)	12 (85.7)	
Humerus	5	4 (80.0)	1 (20.0)	
Fibula	3	1 (33.3)	2 (66.7)	
Others	3	1 (33.3)	2 (66.7)	
MSTS score (for osteosarcoma) mean [SD]	27.3 [3.6]	26.5 [3.9]	28.0 [3.3]	0.135^c^
Relapse				0.439^b^
No	104	40 (38.5)	64 (61.5)	
Yes	12	6 (50.0)	6 (50.0)	
Body mass index mean [SD]	21.7 [3.9]	22.6 [4.9]	21.0 [2.8]	0.052^c^
Underweight	16	8 (50.0)	8 (50.0)	0.061^b^
Normal	73	23 (31.5)	50 (68.5)	
Overweight/obese	27	15 (55.6)	12 (44.4)	
CHC				0.585^b^
No	60	23 (38.3)	37 (61.7)	
Yes	53	23 (43.4)	30 (56.6)	
Treatment characteristics				
Chemotherapy				0.872^b^
No	21	8 (38.1)	13 (61.9)	
Yes	95	38 (40.0)	57 (60.0)	
Radiation				0.846^b^
No	78	31 (39.7)	47 (60.3)	
Yes	36	15 (41.7)	21 (58.3)	
Surgery				0.099^b^
No	4	0	4 (100)	
Yes	112	46 (41.1)	66 (58.9)	0.145^b^, ^d^
Amputation	3	3 (100)	0	
Resection with allograph reconstruction	20	8 (40.0)	12 (60.0)	
Resection with prosthesis	40	18 (45.0)	22 (55.0)	
Resection (other sites)	49	17 (34.7)	32 (65.3)	
Lobectomy due to metastatic tumor	11	7 (63.6)	4 (36.4)	
Treatment combinations				0.683^b^
Surgery only	14	5 (35.7)	9 (64.3)	
Surgery and chemotherapy	63	26 (41.3)	37 (58.7)	
Surgery, chemotherapy, and radiation	27	12 (44.4)	15 (55.6)	
Others	12	3 (25.0)	9 (75.0)	
Behavior characteristics				
Family functioning mean [SD]	89.1 [14.8]	92.5 [13.1]	86.8 [15.4]	**0.040** ^c^
Physical activity mean [SD]	4.3 [2.8]	4.0 [2.8]	4.6 [2.8]	0.253^c^
Low level (0 to 3 points)	50	24 (48.0)	26 (52.0)	0.220^b^
Moderate level (4 to 6 points)	40	12 (30.0)	28 (70.0)	
High level (7 to 10 points)	26	10 (38.5)	16 (61.5)	

*Note:* Boldface indicates statistical significance at *p* < 0.05.

Abbreviations: CHC, chronic health condition; MSTS, Musculoskeletal Tumor Society; N.A., Not applicable (comparison was not conducted due to the small sample size); PNET, primitive neuro‐ectodermal tumor.

^a^ The proportions (%) represent the proportion of survivors who expressed satisfaction and dissatisfaction within each subgroup (i.e., percentages in each row add up to 100%).

^b^ The chi‐square test was conducted to compare the proportion of the dissatisfied group versus the satisfied group for each variable of interest.

^c^ The Mann–Whitney *U*‐test was conducted to compare the variable of interest (continuous scale) among the dissatisfied group versus the satisfied group.

^d^ The chi‐square test was conducted among individuals who received amputation versus resection with allograph versus resection with prosthesis.

### 3.2 Body Image Perception

**FIGURE 1:**
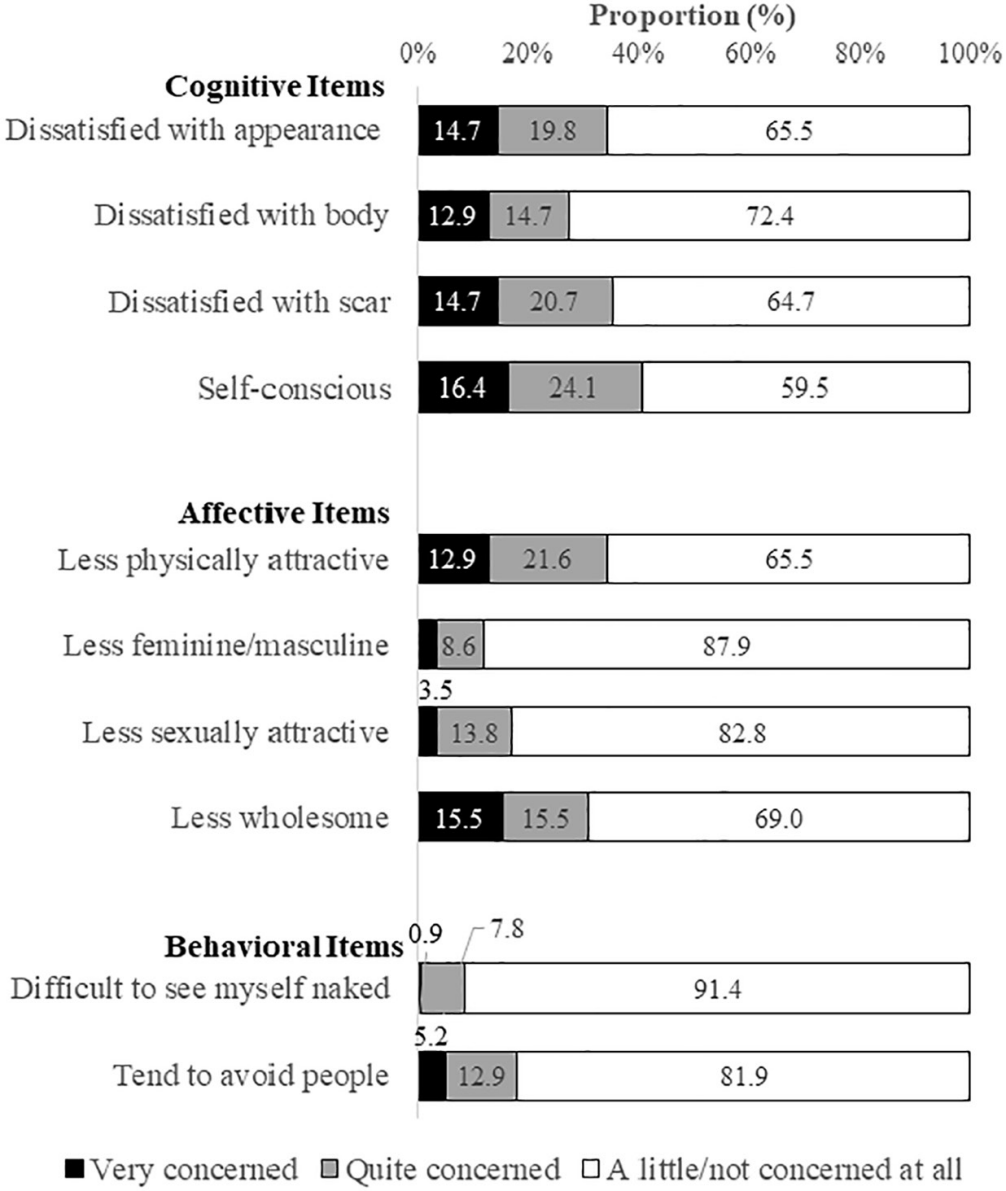
Survivors' body image perception. The body image scale consists of three domains, including the cognitive, affective, and behavioral items.

The median BIS score was 8 (range = 4–14), and the mean score was 9.4 (SD = 7.1). Based on the published cutoff (BIS ≥ 10), the prevalence of body image dissatisfaction was 39.7%. Details of the participants' body image perception are presented in Figure 1. Approximately one‐third of the survivors reported dissatisfaction with their appearance. For cognitive items, 40.5% reported that they felt “self‐conscious” about their appearance. Among the affective items, survivors reported most concern about being “less physically attractive” (34.5%) and “less wholesome” (31%). As for behavioral items, only 8.7% of the survivors were quite or very concerned about “seeing themselves naked.”

### 3.3 Factors Associated With Body Image Dissatisfaction

The demographic, clinical, treatment, and behavioral characteristics of survivors who were satisfied and dissatisfied with their body image are compared in Table 1. Compared with survivors who were satisfied with their body image, survivors who were dissatisfied were older at interview (mean = 30.8 years, SD = 7.3 years versus mean = 26.5 years, SD = 8.3 years, respectively; *p* = 0.005). Survivors with body image dissatisfaction also had a higher BMI than those who reported satisfaction (mean = 22.6, SD = 4.9 kg/m^2^ versus mean = 21.0, SD = 2.8 kg/m^2^; *p* = 0.052). Survivors with body image dissatisfaction reported higher total CFAI scores (mean = 92.5, SD = 13.1 versus mean = 86.8, SD = 15.4; *p* = 0.040), indicating relatively poor family functioning in the dissatisfied group. There was a higher proportion of survivors that were employed full‐time who reported body image dissatisfaction than survivors who were not employed or were still schooling (49.4% versus 17.1%; *p* = 0.001). The two groups did not significantly differ in terms of subtype of sarcoma, level of physical activity, or MSTS score (for survivors with osteosarcoma only).

The multivariable analyses showed that the significant risk factors predicting a poorer total score on the BIS (as a continuous variable) were being female (Est = 3.01, SE = 1.49; *p* = 0.047), surgery treatment (Est = 7.41, SE = 3.65; *p* = 0.046), and worse family functioning (Est = 0.13, SE = 0.05, *p* = 0.013) (Table 2). When comparing the BIS total score in four groups of survivors with different surgery sites, the three survivors who had undergone amputation reported poorer body image perception (BIS mean score = 19.3, SD = 4.0) than those who had received other types of surgeries (Table S2), though this finding should be interpreted cautiously due to the small sample size of patients who received amputation.

**TABLE 2:** Univariate and multivariable analysis of factors associated with body image perception.

Variables	Univariate analysis^a^			Multivariable analysis^a^		
	Est	SE	*p*	Est	SE	*p*
Age at interview (years)^c^	0.28	0.090	**0.003**	0.26	0.144	0.071
Age at diagnosis (years)^c^	0.13	0.093	0.170	0.30	0.155	0.057
Sex						
Male	Ref.	—	—	—	—	—
Female	0.98	0.093	0.297	0.21	0.105	**0.047**
Cancer diagnosis						
Soft‐tissue sarcoma	Ref.	—	—	Ref.	—	—
Osteosarcoma	−0.20	0.092	**0.033**	−0.27	0.134	0.050
Surgery						
No	Ref.	—	—	Ref.	—	—
Yes	0.16	0.092	0.082	0.19	0.094	**0.046**
Radiation						
No	Ref.	—	—	Ref.	—	—
Yes	−0.04	0.095	0.651	0.19	0.131	0.148
Body mass index (kg/m^2^)	0.15	0.110	0.174	0.17	0.110	0.129
Employment^b^						
Unemployed/students	Ref.	—	—	Ref.	—	—
Employed	0.27	0.090	**0.003**	0.08	0.123	0.517
Family functioning (score)	0.21	0.091	**0.021**	0.27	0.106	**0.013**

*Note:* Boldface indicates statistical significance at *p* < 0.05.

Abbreviations: Est, standardized coefficient estimate; SE, standard error.

^a^ The Body Image Scale total score was the dependent continuous variable; patient characteristics were defined as independent variables in the univariate and multivariable regression.

^b^ Employment was categorized as a binary variable: employed versus unemployed/students.

^c^ Age at interview and age at diagnosis are highly correlated. The models for these two variables were run separately, keeping all other variables consistent, to avoid multicollinearity.

### 3.4 Association Between Body Image Perception and Behavioral Outcomes

There were subgroups of survivors who reported depressive problems (13.8%), anxiety (4.3%), somatic problems (6.0%), avoidant personality problems (6.9%), AD/H problems (6.0%), and antisocial problems (6.0%). The mean scores and impairment rates for behavioral measures in the study cohort, and in participants who reported dissatisfaction or satisfaction with their body image perception, are shown in Table S3.

After adjusting for age at interview, sex, type of cancer diagnosis, treatment modalities and weight status, the multivariate analyses (Table 3) showed that poorer body image perception (total BIS score) was significantly associated with more depressive problems (Est = 0.30, SE = 0.103; *p* = 0.005), anxiety problems (Est = 0.36, SE = 0.115; *p* = 0.002), and avoidant personality problems (Est = 0.37, SE = 0.106; *p* < 0.001). The results of the subgroup analysis among survivors of osteosarcoma were consistent with the primary analysis when MSTS score was added as a covariate in the multivariable model (Table S4).

**TABLE 3:** Multivariable analysis of the association between body image perception and behavioral outcomes.

	Body image scale total score^a^		
	Est	SE	*p*
Depressive problems^b^	0.30	0.103	**0.005**
Anxiety problems^b^	0.36	0.115	**0.002**
Somatic problems^b^	0.21	0.114	0.066
Avoidant personality problems^b^	0.37	0.106	**< 0.001**
Antisocial personality^b^	0.15	0.115	0.206

*Note:* All models were adjusted for age, sex, cancer diagnosis, surgery, radiation, and weight status. Boldface indicates statistical significance at *p* < 0.05.

Abbreviations: Est, standardized coefficient estimate; SE, standard error.

^a^ Body Image Scale total score was the continuous independent variable.

^b^ Behavioral outcomes were the dependent variables. A higher score was indicative of worse functioning.

### 3.5 Exploratory Analysis: Examining the Mediating Effects of Body Image Perception

The multivariable analysis has previously identified surgery treatment and family functioning as factors that were significantly associated with body image (Table 2). We explored the mediating effect of body image on the relationship between surgery and behavioral outcomes, and between family functioning and behavioral outcomes, in separate models. The models were controlled for age, sex, cancer diagnosis, radiation, and weight status.

Family functioning was significantly associated with depressive symptoms indirectly through body image (total indirect effect *ß* = 0.12, 95% BCCI 0.07–0.23; proportion‐mediated 20.1%, *p* = 0.043). Similar results were obtained for avoidant personality problems (total indirect effect *ß* = 0.18, 95% BCCI 0.08–0.22; proportion‐mediated 26.3%, *p* = 0.038). Body image was not identified as a mediator between surgery treatment and behavioral outcomes (Table S5).

## 4 Discussion

This is the first study investigating body image dissatisfaction and its associated factors among Chinese survivors of sarcoma. Over one‐third of the Chinese AYA survivors of sarcoma in the study reported dissatisfaction with their body image. Female survivors and those who had undergone surgery were more likely to have concerns about their appearance. Importantly, poor body image perception was associated with more significant internalizing symptoms of depression, anxiety, and avoidant personality. Our results also suggest that body image dissatisfaction mediates the association between poor family functioning and psychological distress among adult survivors. Taken together, body image can be considered a potential modifiable risk factor to address psychological symptoms in the Chinese population, which could inform future psychological interventions focused on improving body image perception among AYA survivors with sarcoma.

We found that one‐third of the survivors (39.7%) were not satisfied with their body image. In the literature, the proportion of body image dissatisfaction ranges from 13% to 28% among patients with head and neck cancer, breast cancer, and brain tumors in North America and Europe [4, 6, 42, 59]. However, relatively few studies have been conducted on Asian populations. One Korean study reported that 39.3% of survivors of hematological malignancies reported body image dissatisfaction [60]. In addition to the differences in patient characteristics (e.g., age at assessment, cancer diagnoses, time since cancer diagnosis and surgical sites) between our study cohort and the targeted populations from studies in the literature, cultural differences play an important role in disparities in body image perception, as perceptions of body shape and beauty are often influenced by social expectations and societal norms. A cross‐cultural study found more body image concerns among both female and male adolescents from Malaysia and China than those from Australia, particularly regarding weight, body shape, hair, and extremities [61, 62]. For example, normal‐weight and underweight Asian women had a tendency to view themselves as overweight [63, 64]. Our univariate analysis also found a larger proportion of overweight/obese individuals in the dissatisfied group than in the satisfied group. Applying the study findings to the local context, encouraging survivors to engage in existing exercise programs offered by local non‐governmental organizations and academic institutions may also improve survivors' self‐esteem and body image.

Body image concerns may be more relevant to survivors of sarcoma compared with other types of cancer because they typically receive extensive resection of tumor sites in parts of the body that are visible. We found that survivors who had undergone amputation reported poorer body image perception than those who had received other types of surgeries. Fortunately, amputation is less common now as compared to decades ago and most patients with osteosarcoma in upper or lower extremities can now be treated with limb‐salvage surgery [65, 66]. However, the use of a prosthesis may still affect limb functioning, resulting in limited movement and disfiguration. Importantly, we did not find significant differences in body image perception among osteosarcoma survivors who had received tumor resection with allograft reconstruction versus prosthesis, and studies have shown that the former approach is associated with substantially improved functional outcomes [67, 68]. Our findings reinforce the need for holistic care for survivors of sarcoma; in addition to examining the health status and functional limitations of sarcoma survivors, long‐term follow‐up consultations should include questions about body image and mobility to better identify survivors who need psychological assistance.

Participants with body image dissatisfaction demonstrated worse depression, anxiety, and avoidant personality problems than those who did not express concerns about their body image. This finding suggests an association between negative body image perception and internalizing problems in survivors; future studies with prospective assessments at multiple timepoints of the cancer care continuum are needed to investigate the causal relationship between these two constructs. AYA patients may experience negative emotional reactions due to unexpected cancer‐related body changes [69]. Body image dissatisfaction was associated with internalizing behavioral symptoms, suggesting that many patients have a distorted perception of their appearance that manifests as avoidance and social anxiety even years after completion of cancer treatment. Our exploratory analysis did not identify body image perception as a significant mediator between surgery treatment and psychological distress, probably because there are other factors that can affect the surgical experience that were not investigated in our study, such as chronic pain and social support [70, 71, 72]. However, the intrinsic thoughts and beliefs that patients hold about their body and their subjective feelings about their appearance may affect the relationship between the cancer experience and emotional distress. Future work should explore the mediating role of body image in illness perception and resilience, and implement interventions early, before distress develops into significant psychological problems that affect patients' functional outcomes.

Body image dissatisfaction was associated with avoidant personality problems, suggesting worse interpersonal functioning in survivors who expressed body image shame or embarrassment. We also found that negative body image perception mediated the association between poor family functioning and depressive symptoms, as well as avoidance personality. This reinforces the importance of family support and family dynamics in the development of self‐perception in adolescents and young adults [15]. Interestingly, one study reported that among the different sources of social support (family, friends, and significant others), only support from friends significantly mediated the association between body image distress and depressive symptoms in patients with cancer [73]. Peer support groups allow survivors to openly share, discuss, and encourage each other for resilience and self‐acceptance, which can further help reduce appearance‐related anxiety [74]. Collectively, these findings support leveraging survivors' existing social network to improve their perception of body image and their interpersonal relationships. For example, adolescent survivors of osteosarcoma often need special assistance when they return to school after treatment [75]; peer support and buddy programs may help to provide a supportive social environment and improve mental health outcomes as patients transition into survivorship.

Our study has several limitations. First, causal and temporal relationships between body image perception and behavioral symptoms cannot be established, due to the cross‐sectional design of the study. It is likely that body image perception and behavioral symptoms co‐exist and can interact with each other. The mechanism of associations between body image dissatisfaction and behavioral outcomes requires prospective investigation throughout the cancer care continuum from diagnosis to long‐term survivorship. Second, our results are limited by the absence of a non‐cancer control group. However, the impact of cancer on body image perception has been extensively reported in the literature [76]. Therefore, the risk factor analysis and the associations between body image and behavioral outcomes might still be valid and applicable to the population of AYA cancer survivors. Third, our study population was small and participants were recruited from a single public hospital. However, survivors of osteosarcoma and STS who are treated in the public healthcare system in Hong Kong typically receive standard treatment protocols that are similar to other developed countries. Hence, it is reasonable to assume that our findings might be generalizable to other AYA survivors of sarcoma in Hong Kong, and can also provide a foundation for further studies in other Asian countries or regions.

## 5 Conclusions

Our study provides important perspectives on the evidence of body image distress in young Chinese survivors of sarcoma. Negative perception of body image was associated with depression, anxiety, and avoidant personality problems. Future research should focus on identifying survivors who are at risk of experiencing negative body image perception, especially those who have received radical surgery and reported worse family functioning, and implementing psychological interventions specifically focused on addressing body image dissatisfaction in AYA survivors of sarcoma. The provision of social support and psychosocial intervention early during the cancer care continuum may mitigate the negative impact of body image distress in AYA survivors.

## Author Contributions

**Yihui Wei:** conceptualization (equal), formal analysis (lead), investigation (equal), methodology (equal), writing – original draft (lead), writing – review and editing (equal). **Chung Tin Ma:** conceptualization (supporting), data curation (supporting), formal analysis (supporting), writing – review and editing (supporting). **Michael Can Heng Li:** data curation (lead), formal analysis (supporting), writing – review and editing (supporting). **Keary Rui Zhou:** data curation (equal), writing – review and editing (equal). **Herbert Ho Fung Loong:** conceptualization (equal), investigation (equal), resources (equal), writing – review and editing (equal). **Kwok Chuen Wong:** conceptualization (equal), data curation (equal), investigation (equal), resources (equal), writing – review and editing (equal). **Chi Kong Li:** conceptualization (equal), data curation (equal), investigation (equal), resources (equal), writing – review and editing (equal). **Yin Ting Cheung:** conceptualization (lead), data curation (lead), formal analysis (lead), funding acquisition (lead), investigation (lead), methodology (lead), project administration (lead), resources (lead), supervision (lead), writing – original draft (lead), writing – review and editing (lead).

## Ethics Statement

This is an observational study. The study was approved by the Joint Chinese University of Hong Kong–New Territories East Cluster Clinical Research Ethics Committee (Ref: 2018.131).

## Consent

Informed consent was obtained from all individual participants included in the study.

## Conflicts of Interest

The authors declare no conflicts of interest.

## Supporting information


Data S1.

## Data Availability

The data that support the findings of this study are available on request from the corresponding author. The data are not publicly available due to privacy or ethical restrictions.
