# Supplementary material for: Impact of Body Image Perception on Behavioral Outcomes in Chinese Adolescent and Young Adult Survivors of Sarcoma
Source: Cancer Med. 2024 Dec 2;13(23):e70320. doi: 10.1002/cam4.70320 (PMC11612020; doi:10.1002/cam4.70320)
Supplement: Supplementary file 1 — Data S1. [file CAM4-13-e70320-s001.docx]

Supplemental Figure 1. Flow Diagram of Recruitment

**Screened**

(n=148)

**Excluded (n=16)**

Non-malignant tumor (n=7)

Developmental or psychiatric disorders before diagnosis of cancer (n=3)

Non-Chinese speaking (n=1)

Currently treated for second cancer or have relapsed (n=5)

**Eligible**

(n=132)

Non-participants (n=16)

Uncontactable (n=2)

Relocated to another country (n=1)

Defaulted appointment (n=4)

Refusal (did not have time) (n=3)

Refusal (not interested) (n=4)

Withdrawal (did not have time to finish) (n=2)

Response rate: 88%

**Completed all assessments**

(n=116)

**Analyzed**

(n=116)

Supplemental Table 1. Reliability and Item-to-Scale Correlation of the Traditional Chinese version of the Body Image Scale

| Item number | Item details | Corrected item-to-scale correlation^+^ | Cronbach’s α |
| --- | --- | --- | --- |
|  | **Overall scale** |  | 0.91 |
|  | **Affective items** |  | 0.85 |
| 2 | Have you felt less physically attractive as a result of your disease or treatment? | 0.82 |  |
| 4 | Have you been feeling less feminine/masculine as a result of your disease or treatment? | 0.65 |  |
| 6 | Have you been feeling less sexually attractive as a result of your disease or treatment? | 0.76 |  |
| 8 | Have you been feeling the treatment has left your body less whole? | 0.72 |  |
|  | **Behavioral items** |  | 0.75 |
| 5 | Did you find it difficult to look at yourself naked? | 0.62 |  |
| 7 | Did you avoid people because of the way you felt about your appearance? | 0.72 |  |
|  | **Cognitive items** |  | 0.84 |
| 1 | Have you been feeling self-conscious about your appearance? | 0.69 |  |
| 3 | Have you been dissatisfied with your appearance when dressed? | 0.75 |  |
| 9 | Have you felt dissatisfied with your body? | 0.75 |  |
| 10 | Have you been dissatisfied with the appearance of your scar? | 0.69 |  |

+ Corrected item-to-scale correlation refers to the correlation between each item and the overall scale score that excludes the particular item.

For both corrected item-to-scale correlation coefficients and Cronbach’s α, values greater than 0.7 were considered to be good, between 0.6 and 0.7 as acceptable, and less than 0.6 as weak.

Supplemental Table 2. Classification of Cancer Diagnosis and Body Image Scale Total Score among Survivors Who Received Different Types of Surgery

| Surgery Type | STS  n=55 | Osteosarcoma  n=57 | Overall  n=112 | BIS Total score | |
| --- | --- | --- | --- | --- | --- |
|  | n (%) | n (%) | n (%) | Mean (SD) | *P* ^#^ |
| Amputation | 2 (3.6) | 1 (1.7) | 3 (2.7) | 19.3 (4.0) | 0.067 |
| Resection with allograft | 2 (3.6) | 18 (31.6) | 20 (17.9) | 10.2 (8.1) |  |
| Resection with prosthesis | 6 (10.9) | 34 (59.7) | 40 (35.7) | 10.3 (7.3) |  |
| Resection (other sites) | 45 (81.9) | 4 (7.0) | 49 (43.7) | 8.1 (6.4) |  |

STS: Soft-tissue sarcoma; BIS: Body Image Scale; SD: standard deviation

^#^Comparison among groups was conducted using Kruskal-Wallis test.

The results should be interpreted cautiously due to the small sample size of survivors who received amputation.

Supplemental Table 3. Rates of Behavioral Problems among Survivors Satisfied/Dissatisfied with Body Image

| Behavioral outcomes | Overall  n= 116 | | | Dissatisfied  n= 46 | | | Satisfied  n= 70 | | |  |
| --- | --- | --- | --- | --- | --- | --- | --- | --- | --- | --- |
|  | Mean (SD) ^*^ | Impaired (%) ^#^ | 95% CI | Mean (SD) ^*^ | Impaired (%) ^#^ | 95% CI | Mean (SD) ^*^ | Impaired (%) ^#^ | 95% CI | *P ^^^* |
| Depressive problems | 56.1 (7.7) | 13.8 | 7.4 – 20.2 | 58.6 (7.9) | 21.7 | 9.4 – 34.1 | 54.5 (7.2) | 8.6 | 1.8 – 15.3 | **0.005** |
| Anxiety problems | 53.3 (5.6) | 4.3 | 0.6 – 8.1 | 55.0 (6.4) | 4.3 | 0 – 10.5 | 52.2 (4.8) | 4.3 | 0 – 9.1 | **0.008** |
| Somatic problems | 55.2 (6.3) | 6.0 | 1.6 – 10.4 | 56.8 (7.1) | 6.5 | 0 – 13.9 | 54.1 (5.4) | 5.7 | 0.1 – 11.3 | **0.022** |
| Avoidant personality problems | 55.7 (6.2) | 6.9 | 2.2 – 11.6 | 57.8 (7.1) | 15.2 | 4.4 – 26.0 | 54.4 (5.1) | 1.4 | 0 – 4.3 | **0.003** |
| Antisocial personality | 53.5 (5.7) | 6.0 | 1.6 – 10.4 | 53.3 (5.1) | 4.3 | 0 – 10.5 | 53.7 (6.1) | 7.1 | 0.1 – 13.3 | 0.760 |

SD: standard deviation; CI: conference interval.

^*^The behavioral measures were transformed into age-adjusted *T*-scores (mean = 50; SD = 10) using population norms. A higher score was indicative of worse functioning or more severe problems.

^#^To estimate the prevalence of impairments within the study samples, impairment was defined as a *T*-score of > 65 (i.e. 1.5 SD worse than the population norms).

^^^Comparison of T-score was conducted using t-test between dissatisfied group and satisfied group.

Supplemental Table 4. Multivariable Analysis of the Association between Body Image Perception and Behavioral Outcomes among Survivors with Osteosarcoma

|  | **Body Image Scale Total Score^#^** | | |
| --- | --- | --- | --- |
|  | **Est** | **SE** | **P** |
| **Depressive Problems^*^** | 0.40 | 0.101 | **<0.001** |
| **Anxiety Problems^*^** | 0.56 | 0.159 | **0.001** |
| **Somatic Problems^*^** | 0.28 | 0.154 | 0.076 |
| **Avoidant Personality Problems^*^** | 0.57 | 0.128 | **<0.001** |
| **Antisocial Personality^*^** | 0.09 | 0.135 | 0.510 |

Est: standardized coefficient estimate; SE: standard error.

^#^ Body Image Scale total score was the continuous independent variable.

^*^ Behavioral outcomes were the dependent variables. A higher score was indicative of worse functioning.

Multivariable analyses were conducted among survivors with osteosarcoma (n=57). All models were adjusted for age, sex, cancer diagnosis, surgery, radiation, weight status and Musculoskeletal Tumor Society score. Boldface indicates statistical significance at *P*<0.05.

Supplemental Table 5. Exploratory Mediation Analysis

|  | Outcome | Marginal Total Effect* | Controlled Direct Effect* | Total Indirect Effect* | Proportion Mediated (%) | *P* |
| --- | --- | --- | --- | --- | --- | --- |
| Family functioning | Depressive problems | 0.31 (0.15, 0.48) | 0.19 (0.11, 0.26) | 0.04 (0.01, 0.09) | 20.1 | **0.043** |
|  | Anxiety problems | 0.26 (0.13, 0.32) | 0.14 (0.11, 0.18) | 0.03 (0.01, 0.06) | 36.7 | 0.14 |
|  | Avoidant personality problems | 0.36 (0.14, 0.48) | 0.19 (0.11, 0.25) | 0.04 (0.02, 0.07) | 26.3 | **0.038** |
| Surgery | Depressive problems | 5.09 (-1.77, 11.95) | 2.47 (-4.12, 9.06) | 1.75 (-0.44, 5.40) | 15.3 | 0.46 |
|  | Anxiety problems | 3.25 (-1.74, 8.25) | 1.36 (-3.44, 6.17) | 1.29 (-0.51, 2.98) | 23.1 | 0.21 |
|  | Avoidant personality problems | 2.87 (-2.69, 8.43) | 1.13 (-4.32, 6.59) | 1.25 (-0.92, 3.80) | 15.7 | 0.32 |

* Bias corrected confidence intervals for mediation analysis in which body image was represented as mediators in the association between the independent variables (i.e. family functioning and surgery treatment) and behavioral outcomes (i.e. depressive problems, anxiety problems, and avoidant personality problems). Both models were controlled for age, sex, cancer diagnosis, radiation, and weight status.
